# Cutaneous Leishmaniasis Outbreak Investigation in Hajjah Governorate, Yemen, in 2018: Case-Control Study

**DOI:** 10.2196/27442

**Published:** 2021-05-14

**Authors:** Abdulkareem Ali Nassar, Mahmood Hasan Abdelrazzaq, Ali Hamoud Almahaqri, Mohammed Abdullah Al-Amad, Abulwahed Abduljabbar Al Serouri, Yousef Saleh Khader

**Affiliations:** 1 Sana'a City's Public Health and Population Office Yemen Ministry of Public Health and Population Sana'a Yemen; 2 Noncommunicable Diseases World Health Organization Sana'a Yemen; 3 Leishmaniasis Control Program Yemen Ministry of Public Health and Population Sana'a Yemen; 4 Field Epidemiology Training Program Yemen Ministry of Public Health and Population Sana'a Yemen; 5 Department of Public Health, Community Medicine and Family Medicine Faculty of Medicine Jordan University of Science & Technology Amman Jordan

**Keywords:** cutaneous leishmaniasis, outbreak, risk factors, Yemen, Field Epidemiology Training Program

## Abstract

**Background:**

Cutaneous leishmaniasis (CL) is endemic in Yemen. About 4440 cases were reported in 2019. On July 23, 2018, a Hajjah governorate surveillance officer notified the Ministry of Public Health and Population about an increase in the number of CL cases in Bani-Oshb, Kuhlan district, Hajjah governorate. On July 24, 2018, Yemen Field Epidemiology Training Program sent a team to perform an investigation.

**Objective:**

We aimed to describe a CL outbreak in Hajjah governorate and determine its risk factors.

**Methods:**

A descriptive study and case-control study (1:1 ratio) were conducted. Cases included people who met the suspected or confirmed case definition of the World Health Organization and lived in Bani-Oshb subdistrict during the period from August 2017 to July 2018. Controls included people living for at least 1 year in Bani-Oshb without new or old skin lesions. Crude odds ratios (cORs) and adjusted odds ratios (aORs) with 95% CI were used to test the significance of associations.

**Results:**

We identified 30 CL cases. Among the 30 patients, 7 (23%) were younger than 5 years, 17 (57%) were 5 to 14 years, 17 (57%) were females, and 23 (77%) had one lesion. The attack rate was 7 per 1000 population in the age group <15 years and 1 per 1000 population in the age group ≥15 years. On bivariate analysis, the following factors were significantly associated with CL: female gender (cOR 5.2, 95% CI 1.7-16.5), malnutrition (cOR 5.2, 95% CI 1.7-16.5), not using a bed net (cOR 14.5, 95% CI 1.7-122.4), poor house lighting (cOR 6.4, 95% CI 2.1-19.7), poor house hygiene (cOR 11.2, 95% CI 3.1-40.7), poor sanitation (cOR 14.5, 95% CI 1.7-122.4), living in houses without window nets (cOR 5.2, 95% CI 1.3-21.2), plantation around the house (cOR 6.5, 95% CI 2.1-20.5), animal barn inside or close to the house (cOR 9.3, 95% CI 1.9-46.7), raising animals (cOR 8.1, 95% CI 1.6-40.7), and having animal dung in or near the house (cOR 6.8, 95% CI 1.7-27.7). The following risk factors remained significant on multivariate stepwise analysis: female gender (aOR 22.7, 95% CI 1.6-320.5), malnutrition (aOR 17.2, 95% CI 1.3-225.8), poor house hygiene (aOR 45.6, 95% CI 2.5-846.4), plantation around the house (aOR 43.8, 95% CI 1.9-1009.9), and raising animals (aOR 287.1, 95% CI 5.4-15205.6).

**Conclusions:**

CL was endemic in Hajjah governorate, and an increase in cases was confirmed. Many individual, housing, and animal related factors were shown to contribute to CL endemicity. Implementation of control measures directed toward altering the factors favoring contact among vectors, reservoirs, and susceptible humans is strongly recommended to control future outbreaks.

## Introduction

Leishmaniasis is a parasitic disease that is found in parts of the tropics, subtropics, and southern Europe. It is caused by infection with *Leishmania* parasites, which are spread by the bite of infected sand flies [1,2]. There are different forms of leishmaniasis. The most common form is cutaneous leishmaniasis (CL), which causes skin sores [1]. Most people have CL without any symptoms or signs [3]. People who develop clinical evidence of infection have one or more sores on their skin. The sores can change in size and appearance over time. The sores may start as nodules and may end up as ulcers like a volcano, with a raised edge and central crater [1].

Globally, approximately 700,000 to 1 million new cases and 26,000 to 65,000 deaths occur annually [4,5]. The disease is associated with malnutrition, population displacement, poor housing, a weak immune system, and poor socioeconomic status [4]. In the Eastern Mediterranean Region (EMR), it has been reported in many countries, including Afghanistan, Iran, Iraq, Pakistan, and Syria [4]. The EMR accounts for 70% of the CL cases worldwide [4].

CL is endemic in the north-western region of Yemen [6-8]. About 4440 CL cases were reported in 2019 [9]. On July 23, 2018, a Hajjah governorate surveillance officer notified the Ministry of Public Health and Population about an increase in the number of CL cases in Bani-Oshb, Kuhlan district, Hajjah governorate. On July 24, 2018, Yemen Field Epidemiology Training Program sent a team to perform an investigation.

This study aimed to confirm the existence of a CL outbreak in Bani-Oshb, describe the characteristics of CL by person, place, and time, and determine the risk factors of a CL outbreak.

## Methods

### Study Area

This study was conducted in Bani-Oshb subdistrict in Kuhlan Affar district, Hajjah governorate, Yemen. It has a population of 7453 persons (The Immunization Department of The Public Health and Population Office, Kuhlan Affar district, unpublished data, 2017). It has only one health unit, but it is not functional. It is rainy in summer and cold and dry in winter. Most people in Bani-Oshb have cattle and farms. They depend on raising animals and agricultural activities, and their houses are surrounded by farms and trees.

### Study Design

This investigation consisted of descriptive and analytic studies. A line list was developed to collect data, and an active house-to-house search was performed. The World Health Organization (WHO) case definition for CL was used [10]. A suspected case was defined as the presence of clinical signs (skin lesions) without parasitological confirmation of the diagnosis. A confirmed case was defined as the presence of clinical signs with parasitological confirmation of the diagnosis (positive smear or culture from a skin lesion).

A case-control study design was used to determine the risk factors associated with CL (30 cases and 30 controls). Cases included people who met the suspected or confirmed case definition of the WHO and lived in Bani-Oshb subdistrict during the period from August 2017 to July 2018. Controls included people living in the house without any recorded new or old skin lesions among house members according to the household and examination of a medical doctor, and living for at least 1 year in Bani-Oshb without new or old skin lesions.

### Data Collection and Diagnosis

A structured questionnaire was used to collect data on individual, housing, and animal related characteristics. Data were collected on different variables, including age, gender, use of bed nets, house lighting, house hygiene (daily routine of cleaning rooms and disposal of garbage), sanitation (presence of a latrine with safe disposal of human waste), plantation around the house, animal barn inside or close to the house, raising of animals, and animal dung in or near the house. The data on these variables were self-reported by the participants or obtained by observation. One member of the research team collected 22 skin scrapings (small quantities of tissue) from cases. The tissues were directly smeared on glass slides, air dried, and fixed with methanol for a few seconds. After 20 minutes of staining, the slides were washed with water and left to dry in air. Then, the stained smears were sent to the National Central Public Health Laboratories. The laboratory result was considered positive if an amastigote was seen or negative if an amastigote was not seen after 15 minutes of inspection.

### Data Analysis

Data were analyzed using Epi Info version 7.2 [11]. Data were described using percentages. Crude odds ratios (cORs) or adjusted odds ratios (aORs) with 95% CIs were used to test the significance of associations in bivariate and multivariate stepwise analyses. A *P* value <.05 was considered statistically significant.

## Results

### Patient Characteristics

**Figure 1:**
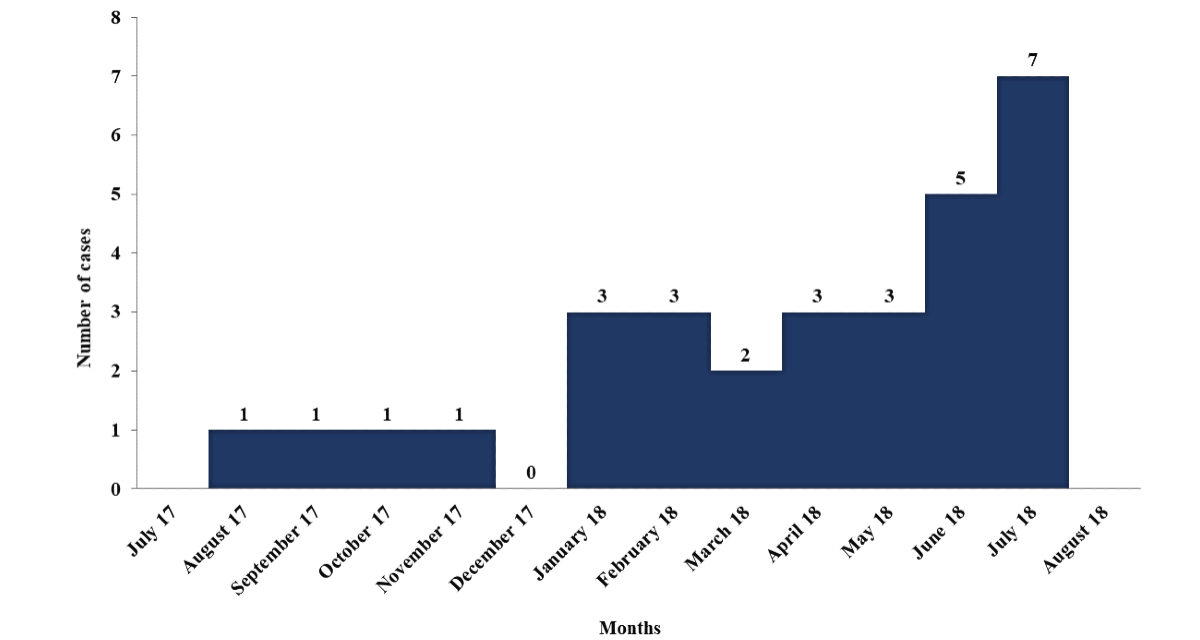
The distribution of cutaneous leishmaniasis cases by time in Bani-Oshb, Hajjah governorate from August 2017 to July 2018 (N=30).

A total of 30 cases of leishmaniasis were found in Bani-Oshb subdistrict during the period from August 2017 to July 2018. The number of cases started to increase in January 2018 and reached a peak in July 2018 (Figure 1).

Table 1 shows the characteristics of the CL cases. Of the 30 patients, 7 (23%) were younger than 5 years, 17 (57%) were aged 5 to 14 years, and 6 (20%) were older than 14 years. Additionally, females represented 17 (57%) cases. Moreover, 23 (76%) patients had one lesion, 4 (14%) had two to three lesions, and 3 (10%) had more than three lesions. The face was the most affected part of the body (20/30, 67%), followed by the lower limbs (4/30, 13%) and upper limbs (3/30, 10%). In 10% (3/30) of cases, both the face and limbs were affected. Bait Rokn and Bait Alfrwi were the most affected villages with 12 (40%) cases. The attack rate was 7 per 1000 population in the age group <15 years and 1 per 1000 population in the age group ≥15 years. Among 22 skin scraping samples, 21 (96%) were positive, and leishmaniasis amastigotes were seen.

**Table 1:** Characteristics of cutaneous leishmaniasis cases (N=30).

Characteristic			Value, n (%)
**Age (years)**			
	<5	7 (23%)	
	5-14	17 (57%)	
	≥15	6 (20%)	
**Sex**			
	Female	17 (57%)	
	Male	13 (43%)	
**Village of residence**			
	Bait Rokn	6 (20%)	
	Bait Alfrwi	6 (20%)	
	Bait Joma'an	4 (13%)	
	Al Wadi	3 (10%)	
	Fra'ah	3 (10%)	
	Al Obal	2 (7%)	
	Bani Bram	2 (7%)	
	Bait Za'abl	2 (7%)	
	Bait Alwali	1 (3%)	
	Arshan	1 (3%)	
**Number of lesions**			
	1	23 (76%)	
	2	2 (7%)	
	3	2 (7%)	
	>3	3 (10%)	
**Site of lesions**			
	Face	20 (67%)	
	Lower limbs	4 (13%)	
	Upper limbs	3 (10%)	
	Face and limbs	3 (10%)	

### Factors Associated With CL

Table 2 shows the bivariate analysis for associated risk factors of CL. Female gender (cOR 5.2, 95% CI 1.7-16.5), malnutrition (cOR 5.2, 95% CI 1.7-16.5), and not using a bed net (cOR 14.5, 95% CI 1.7-122.4) were associated with increased odds of CL. Poor house lighting (cOR 6.4, 95% CI 2.1-19.7), poor house hygiene (cOR 11.2, 95% CI 3.1-40.7), and poor sanitation (cOR 14.5, 95% CI 1.7-122.4) were associated with higher odds of CL. Moreover, other risk factors were identified, including living in houses without window nets (cOR 5.2, 95% CI 1.3-21.2), plantation around the house (cOR 6.5, 95% CI 2.1-20.5), animal barn inside or close to the house (cOR 9.3, 95% CI 1.9-46.7), raising animals (cOR 8.1, 95% CI 1.6-40.7), and having animal dung in or near the house (cOR 6.8, 95% CI 1.7-27.7).

The following risk factors remained significant on multivariate stepwise analysis (Table 3): female gender (aOR 22.7, 95% CI 1.6-320.5), malnutrition (aOR 17.2, 95% CI 1.3-225.8), poor house hygiene (aOR 45.6, 95% CI 2.5-846.4), plantation around the house (aOR 43.8, 95% CI 1.9-1009.9), and raising animals (aOR 287.1, 95% CI 5.4-15205.6).

**Table 2:** Bivariate analysis of associated risk factors of cutaneous leishmaniasis in Bani-Oshb, Hajjah governorate from August 2017 to July 2018.

Risk factor		Cases (n=30), n (%)	Controls (n=30), n (%)	cOR^a^ (95% CI)	*P* value
**Age (years)**				0.8 (0.2-2.9)	.74
	<15	24 (80%)	25 (83%)		
	≥15	6 (20%)	5 (17%)		
**Sex**				5.2 (1.7-16.5)	.003
	Female	17 (57%)	6 (20%)		
	Male	13 (43%)	24 (80%)		
**Malnutrition**				5.2 (1.7-16.5)	.003
	Yes	24 (80%)	13 (43%)		
	No	6 (20%)	17 (57%)		
**Bed net use**				14.5 (1.7-122.4)	.002
	Never	29 (97%)	20 (67%)		
	Use	1 (3%)	10 (33%)		
**House lighting**				6.4 (2.1-19.7)	<.001
	Poor	22 (73%)	9 (30%)		
	Good	8 (27%)	21 (70%)		
**House hygiene**				11.2 (3.1-40.7)	<.001
	Poor	26 (87%)	11 (37%)		
	Good	4 (13%)	19 (63%)		
**Sanitation**				14.5 (1.7-122.4)	.003
	Poor	29 (97%)	20 (67%)		
	Good	1 (3%)	10 (33%)		
**Windows of the house**				5.2 (1.3-21.2)	.02
	Without net	27 (90%)	19 (63%)		
	With net	3 (10%)	11 (37%)		
**Plantation around the house**				6.5 (2.1-20.5)	<.001
	Yes	20 (67%)	7 (23%)		
	No	10 (33%)	23 (77%)		
**Animal barn inside or close to the house**				9.3 (1.9-46.7)	.002
	Yes	28 (93%)	18 (60%)		
	No	2 (7%)	12 (40%)		
**Raising animals**				8.1 (1.6-40.7)	.004
	Yes	28 (93%)	19 (63%)		
	No	2 (7%)	11 (37%)		
**Animal dung in or near the house**				6.8 (1.7-27.7)	.003
	Yes	27 (90%)	17 (57%)		
	No	3 (10%)	13 (43%)		

^a^cOR: crude odds ratio.

**Table 3:** Risk factors on multivariate stepwise analysis in Bani-Oshb, Hajjah governorate from August 2017 to July 2018.

Risk factor		Cases (n=30), n (%)	Controls (n=30), n (%)	aOR^a^ (95% CI)	*P* value
**Sex**				22.7 (1.6-320.5)	.02
	Female	17 (57%)	6 (20%)		
	Male	13 (43%)	24 (80%)		
**Malnutrition**				17.2 (1.3-225.8)	.03
	Yes	24 (80%)	13 (43%)		
	No	6 (20%)	17 (57%)		
**House hygiene**				45.6 (2.5-846.4)	.01
	Poor	26 (87%)	11 (37%)		
	Good	4 (13%)	19 (63%)		
**Plantation around the house**				43.8 (1.9-1009.9)	.02
	Yes	20 (67%)	7 (23%)		
	No	10 (33%)	23 (77%)		
**Raising animals**				287.1 (5.4-15205.6)	.005
	Yes	28 (93%)	19 (63%)		
	No	2 (7%)	11 (37%)		

^a^aOR: adjusted odds ratio.

## Discussion

### Principal Findings

A total of 30 cases with leishmaniasis were found in Bani-Oshb subdistrict during the period from August 2017 to July 2018. Our results showed that most CL cases involved patients between the ages of 5 and 14 years and involved females. Additionally, most cases involved one lesion. The face was the most affected part of the body. We also observed that many individual, housing, and animal related factors, such as malnutrition, poor house hygiene, plantation around the house, and raising animals, were significantly associated with CL infection.

The number of cases showed an increase in January and reached a peak in July. This might be due to the rainy season, which is a favorable time for sand fly activity and breeding [8,12]. The age group less than 15 years was the most affected, with an attack rate of 7 per 1000 population. This finding might be explained by the fact that children are usually not aware about how to protect themselves from bites of sand flies. Besides, children spend most of their time outdoors. This finding is similar to the findings in other studies from Taiz and central Yemen governorates, as well as Iran [8,13,14].

Females were more likely to be infected than males. This might be explained by the fact that rural women in Hajjah governorate perform different tasks, including domestic animal care, getting water, and agricultural activities, which increase their probability of exposure to sand fly bites. This finding is consistent with findings in studies from Iran and Lebanon [14,15]. However, this finding disagrees with findings in other studies from Lahj, Hajjah, and Amran [6,7,12].

More than three-quarters of CL cases had one lesion. This finding confirms the findings of previous studies from Lahj, Hajjah, Taiz, and Nepal [6-8,16]. In agreement with the findings of other studies [6,7,12], two-thirds of cases had lesions on their faces, and this might be because the face is the most exposed part of the body.

Malnutrition was found to be significantly associated with increased risk of CL infection, which may be due to increased individual susceptibility to infection with CL. Few studies have confirmed the relationship between malnutrition and risk of CL [17,18].

Not using a bed net was significantly associated with increased risk of CL infection. This might be due to the absence of personal protection against the vector. This finding is consistent with the findings of studies from Ethiopia, Waziristan, and Turkey [5,19,20]. However, it is not consistent with the findings of other studies from Kenya, Bolivia, Thailand, and Afghanistan [2,21-23].

This investigation indicated that there was an association between CL infection and poor housing characteristics, including poor lighting, house hygiene, and sanitation. These findings might be explained by the fact that such conditions are favorable for the activity and breeding of sand flies. Similar findings have been reported in Amran governorate [12]. Moreover, living in houses without window nets was associated with an increased risk of CL, possibly because of increased exposure to sand flies. One study from Afghanistan did not report a significant association between living in houses without windows and CL [23]. Furthermore, there was an association between plantations around the house and CL. This finding is consistent with the findings of studies from Kenya, Amran, Waziristan, Sri Lanka, and Palestine [2,12,19,24,25] and is not consistent with the findings of a study from Thailand [22]. Additionally, having an animal barn inside or close to the house was associated with CL, which has been reported in other studies from Taiz, Amran, and Turkey [8,12,20], but not in a study from Thailand in 2016 [22].

There was an association between animal-related factors and CL infection. Animals seem to be an important reservoir for maintaining the life cycle of many *Leishmania* species and transmission of infection. Similar findings were reported in studies from Taiz, Amran, Ethiopia, Turkey, and Palestine [5,8,12,20,25], but not in a study from Thailand [22]. Furthermore, animal dung in or close to the house increased the risk of CL infection because this creates a favorable environment to attract sand flies into human settlements. This finding agreed with the findings of a study from Turkey [20]. 

However, some of the abovementioned significant risk factors identified in bivariate analysis, which were also reported in previous studies, did not remain significant on multivariate stepwise analysis (eg, bed net use, house lighting, and sanitation). This may be because of the small sample size of this study or because those factors were not risk factors in the studied community.

This study has some limitations related to the study design and sample size. Recall bias might be present owing to the retrospective design of this study. The small sample size is possibly the most important limitation, which generated a wider CI. The study was performed in a subdistrict with mountainous terrain where communities are scattered and difficult to reach, and their work in agricultural activities during the day limited participation in this study. Moreover, difficulty in obtaining eligible controls in this endemic area was the main drawback of enrolling more controls.

### Conclusions

CL was endemic in Hajjah governorate, and an increase in cases was confirmed. Many individual, housing, and animal related factors were shown to contribute to CL endemicity. Implementation of control measures directed toward altering the factors favoring contact among vectors, reservoirs, and susceptible humans, such as malnutrition and plantation around the house, is recommended to control future outbreaks. Further studies focusing on the species of parasites, vectors, and reservoirs are recommended.

## Abbreviations

aOR: adjusted odds ratio
cOR: crude odds ratio
CL: cutaneous leishmaniasis
EMR: Eastern Mediterranean Region
WHO: World Health Organization
